# Predictive biological markers of systemic lupus erythematosus flares: a systematic literature review

**DOI:** 10.1186/s13075-017-1442-6

**Published:** 2017-10-24

**Authors:** Noémie Gensous, Aurélie Marti, Thomas Barnetche, Patrick Blanco, Estibaliz Lazaro, Julien Seneschal, Marie-Elise Truchetet, Pierre Duffau, Christophe Richez, on behalf of the FHU ACRONIM

**Affiliations:** 1 ImmunoConcept, UMR CNRS 5164, Université de Bordeaux, Bordeaux, France; 2 Department of Internal Medicine and Clinical Immunology, Saint-Andre Hospital, Bordeaux, France; 3 Department of Dermatology, Saint-Andre Hospital, Bordeaux, France; 4 Department of Rheumatology, Pellegrin Hospital, Place Amélie Raba Léon, 33076 Bordeaux, France; 5 Department of Internal Medicine and Infectious Diseases, Haut-Leveque Hospital, Pessac, France

**Keywords:** Systemic lupus erythematosus, Biomarker, Flare, Exacerbation, Systematic review

## Abstract

**Background:**

The aim of this study was to identify the most reliable biomarkers in the literature that could be used as flare predictors in systemic lupus erythematosus (SLE).

**Methods:**

A systematic review of the literature was performed using two databases (MEDLINE and EMBASE) through April 2015 and congress abstracts from the American College of Rheumatology and the European League Against Rheumatism were reviewed from 2010 to 2014. Two independent reviewers screened titles and abstracts and analysed selected papers in detail, using a specific questionnaire. Reports addressing the relationships between one or more defined biological test(s) and the occurrence of disease exacerbation were included in the systematic review.

**Results:**

From all of the databases, 4668 records were retrieved, of which 69 studies or congress abstracts were selected for the systematic review. The performance of seven types of biomarkers performed routinely in clinical practice and nine types of novel biological markers was evaluated. Despite some encouraging results for anti-double-stranded DNA antibodies, anti-C1q antibodies, B-lymphocyte stimulator and tumour necrosis factor-like weak inducer of apoptosis, none of the biomarkers stood out from the others as a potential gold standard for flare prediction. The results were heterogeneous, and a lack of standardized data prevented us from identifying a powerful biomarker.

**Conclusions:**

No powerful conclusions could be drawn from this systematic review due to a lack of standardized data. Efforts should be undertaken to optimize future research on potential SLE biomarkers to develop validated candidates. Thus, we propose a standardized pattern for future studies.

**Electronic supplementary material:**

The online version of this article (doi:10.1186/s13075-017-1442-6) contains supplementary material, which is available to authorized users.

## Background

Systemic lupus erythematosus (SLE) is a systemic autoimmune disease characterized by a relapsing–remitting course or flare pattern. Flares, defined by an increase in disease activity over a defined amount of time, can be measured using various scores. Flares might lead to substantial organ damage, increasing morbidity and mortality rates and resulting in higher healthcare costs [1]. Flares are unpredictable in frequency and severity. It is important to identify patients at greater risk for flares to follow them up closely, to make early diagnoses and to initiate rapid treatment or even to consider preventive therapies [2]. Because of a better understanding of SLE pathogenesis, an increasing number of biomarkers have emerged. Close relationships of serum, plasma or urinary profiles with the course of the disease have been explored. Since the 1970s, no investigators have succeeded in identifying a biomarker with the potential to predict efficiently the occurrence of new flares, despite great clinical necessity. We conducted a systematic review of the literature to identify all of the data available on biological SLE flare predictors.

## Methods

We registered our protocol in PROSPERO (an international prospective registry of systematic reviews) under registration number CRD42015026141. The systematic review was written in accordance with the Preferred Reporting Items for Systematic Reviews and Meta-Analyses (PRISMA) guidelines [3].

### Search strategy

Two investigators (NG and AM) conducted a systematic hand search of the literature in collaboration with a research librarian (EM) using two electronic databases, Medline and EMBASE, from inception to 30 April 2015. In order to cover any research that was not yet published as a manuscript, congress abstracts of the European League Against Rheumatism (EULAR) and the American College of Rheumatology (ACR) from 2010 to 2014 were considered. The search was restricted to the English and French languages and to human subjects. The search keywords in Medline were: “biological markers”, “lupus erythematosus, systemic”, “severity of illness index”, “flare(s)”, “exacerbation(s)”, “predictive value of tests” and "disease progression". Search terms in EMBASE were: “systemic lupus erythematosus”, “biological markers”, “flare(s)”, “exacerbation(s)”, “predictive value of tests” and “disease progression”. The exact search strategies are provided in Additional file 1. Abstract lists from the EULAR and the ACR were searched using the keyword “systemic lupus erythematosus”. Reference lists of selected papers were hand searched for other relevant publications. We also searched clinicaltrials.gov in May 2015 for unpublished studies on this topic.

### Eligibility criteria and study selection

The two investigators independently screened the titles and abstracts of references, selected articles for full-text review using the inclusion criteria and assessed the methodological quality. Any discrepancies were resolved through consensus. Two supervisors (PD and CR) participated in resolving disagreements.

Interventional studies (randomized or non-randomized, controlled trials) and observational studies (case–control or cohort studies) were included, whereas case reports, literature reviews and editorials were not included. We considered publications involving adults with SLE, addressing the relationships between one or more defined biological test(s) and the occurrence of disease exacerbation. The exclusion criteria were paediatric subjects, other autoimmune diseases and assessment of the role of genetic markers. When duplicate reports were published on the same study, the report with more complete information was extracted.

### Data extraction

The two investigators independently extracted data from each study using a systematic data extraction form (available on request) developed for this specific purpose, including sample size, socio-demographic data and SLE disease characteristics (duration, treatment(s)), follow-up duration and frequency, disease activity measurement (activity indices, definition of flare) and biomarker characteristics (type, measurement method, cut-off values for positivity and increase). After extracting data independently for every study, discrepancies were resolved through consensus.

### Data synthesis

Biomarkers were allocated into one of the following two groups: biomarkers traditionally performed in clinical practice; and experimental and newly developed biological markers. Raw data are available upon request from the first authors.

## Results

**Fig. 1:**
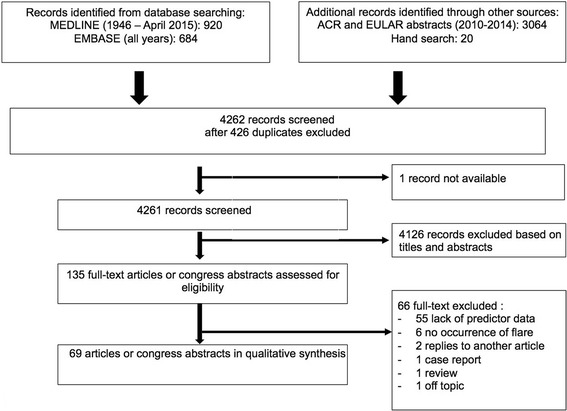
Study selection process. ACR American College of Rheumatology, EULAR European League Against Rheumatism

From both databases, 4668 records were retrieved, and we added 20 studies identified from the reference lists of papers (Fig. 1). A total of 4126 studies failed to meet the required criteria and 135 full-text articles were retained for complete screening. A total of 69 publications were finally included. No ongoing or unpublished trials relative to this topic were found in the www.clinicaltrials.gov database. The detailed characteristics of the included studies appear in Additional file 2.

### Predictors of flares: biomarkers traditionally performed

#### Anti-double-stranded DNA antibodies

From a 1979 study by Swaak et al*.* [4], changes in levels of anti-double-stranded DNA antibodies (anti-dsDNA ab) during the course of the disease were supposed to be related to SLE exacerbations. Table 1 reviews 28 studies, highlighting the major findings [4–31].

**Table 1:** Predictivity of anti-dsDNA antibodies in SLE flares

Assay	Number of patients (*n*)	Findings	Study
Positive results			
CLIF	61	67% of patients in the group with exacerbations had persistent anti-dsDNA ab versus 27% in the patient group without exacerbations	Oelzner et al., 1996 [14]
CLIF	299	Increased level at baseline was a risk factor for flare in the haematological system (*p* = 0.003)	Petri et al., 2009 [10]
CLIF	65	Cases, defined as experiencing a surge in anti-dsDNA from 0 to 3+/4+, or from 1+ to 4+, within a period of less than 12 months, were more likely to experience a severe flare than controls (OR 6.3 (CI 2.0–19.9), *p* = 0.02)	Pan et al., 2014 [25]
Farr	487	Frequency of renal flare was lower in patients with sustained reductions in anti-dsDNA ab (> 10% from baseline levels for at least 2/3 of all observed values) than in patients with stable or increasing antibody levels	Linnik et al., 2005 [24]
Farr	130	All 15 major exacerbations were preceded by an increase of the anti-dsDNA ab levels, with a doubling time of less than 6 weeks for 13 patients. There were four other patients with an increase in anti-dsDNA levels who did not show any exacerbation	Swaak et al., 1982 [11]
Farr	143	A continuous increase in the anti-dsDNA ab was found for all patients in the 24 weeks preceding exacerbations with a doubling time < 10 weeks	Swaak et al., 1986 [12]
Farr	78	A sharp drop in anti-dsDNA ab, usually preceded by a rise, was related to a serious exacerbation	Swaak et al., 1979 [4]
Farr	151	Anti-dsDNA increase started 4 months prior to the relapse and reached a maximum at the moment of relapse whereas no fluctuations were observed in patients with persistently inactive disease	Coremans et al., 1995 [15]
Farr	23	Presence of anti-dsDNA abs (> 5 IU/ml) or increase (> 25%) was associated with a high risk of renal flare	Matrat et al., 2011 [29]
Farr	199	Patients with anti-dsDNA ab (> 15 IU/ml) had a greater risk of developing proliferative glomerulonephritis than patients without auto-antibodies (*p* = 0.048)	Cortés-Hernàndez et al., 2004 [22]
ELISA	70	Anti-dsDNA antibodies were detected in 14 (93.3%) of 15 patients with subsequent lupus nephritis, compared with 24 (72.7%) of 33 patients with active SLE and no nephritis (*p* = ns) and nine (73%) patients with inactive SLE and no nephritis (*p* = ns). Sensitivity for severe lupus nephritis was 100%	Meyer et al., 2009 [30]
Farr and EliA	48	All 22 exacerbations were accompanied by changes in anti-dsDNA (> 25%) in one or both assays	Hillebrand et al., 2013 [26]
CLIF and ELISA	53	Increase in anti-dsDNA ab predicted flares with M-SLEDAI and M-LAI indices	Ho et al., 2001 [17]
CLIF, ELISA and Farr	72	89% of the exacerbations were preceded by a significant increase in anti-dsDNA ab levels (defined as ≥ 2 titres by the *C. luciliae* test or ≥ 25% and at least 100 IU/ml by the ELISA or ≥ 25% and at least 30 IU/ml by the Farr assay)	ter Borg et al., 1990 [13]
ELISA and Farr	34	Patients with rises in IgG class anti-dsDNA ab by ELISA (≥ 6 IU/ml) or in anti-dsDNA by Farr assay (≥ 15 IU/ml) had a significantly higher cumulative risk for relapses, with a median time of 2.3 and 2.1 months respectively	Bootsma et al., 1997 [16]
NA	189	Persistently positive anti-dsDNA after cyclophosphamide treatment was an independent predictor of renal flares	Mok et al., 2004 [23]
NA	218	The combination of complement C3, C4 and anti-dsDNA antibody is reasonably specific for predicting lupus flares in the preceding 4 weeks	To et al., 2011 [28]
NA	562	Elevated titres (≥ 200 IU/ml) at baseline were independent predictors of moderate-to-severe flares at week 52	Petri et al., 2013 [9]
Negative results			
CLIF	27	Serial measurements of anti-dsDNA ab were poor markers of exacerbation.	Lloyd and Schur, 1981 [19]
Farr	48	Changes in anti-dsDNA ab failed to correctly predict a change in disease activity	Abrass et al., 1980 [18]
Farr	202	Fluctuations in anti-dsDNA ab were poor predictors of disease exacerbations according to SLEDAI	Esdaile et al., 1996 [20]
Farr	120	No consistent association between anti-dsDNA ab positivity and risk of flare defined by SLEDAI	Mirzayan et al., 2000 [6]
Farr	46	Baseline anti-dsDNA ab failed to predict renal relapses	El Hachmi et al., 2003 [8]
ELISA	23	Anti-dsDNA ab were not predictive of flare	Steiman et al., 2010 [21]
Farr and CLIF	73	No difference between the patients who flared and the patients who did not	Walz LeBlanc et al., 1994 [31]
NA	57	Percentage of patients who had positive anti-dsDNA ab at the time of the diagnosis was not higher in patients with a subsequent exacerbation	Tomioka et al., 2008 [7]
NA	110	Anti-dsDNA ab were not identified as a predictor of flare	Swaak et al., 1989 [5]
NA	218	Anti-dsDNA lacks sensitivity in predicting serosal and neuropsychiatric lupus flares	To et al., 2011 [27]

*anti-dsDNA ab* anti-double-stranded DNA antibodies, *CI* confidence interval, *CLIF Crithidia luciliae* immunofluorescence, *ELISA* enzyme-linked immunosorbent assay, *EliA* automated enzyme fluoroimmunoassay, *M-LAI* Modified Lupus Activity Index, *M-SLEDAI* Modified Systemic Lupus Erythematosus Disease Activity Index, *NA* not available, *ns* not significant, *OR* odds ratio, *SLE* systemic lupus erythematosus, *SLEDAI* Systemic Lupus Erythematosus Disease Activity Index

Six studies examined anti-dsDNA ab at baseline without follow-up measurements [5–10]. Four studies failed to show any association between baseline anti-dsDNA ab and subsequent flares [5–8]. Two studies of larger size showed that the elevated baseline antibody level was an independent predictor of moderate-to-severe SLE flares (HR = 1.83 (95% confidence interval (CI) 1.29–2.60)) for any new British Isles Lupus Assessment Group (BILAG) A domain at week 52 [9] or a risk factor only for haematologic flares (OR = 2.33 (95% CI 1.34–4.04), *p* = 0.0033) [10].

An increase in anti-dsDNA ab during the course of the disease was found to precede general flares in nine studies [4, 7, 11–17], whereas six studies [6, 18–21, 31] failed to prove such an association.

Interestingly, focusing on renal flares, patients with positive anti-dsDNA ab who had persistent or increasingly levels were at greater risk for subsequent SLE nephritis [22–24].

The results were expressed in terms of sensitivity, specificity and predictive values in six studies [25–30] (Additional file 3). The conclusions were heterogeneous: sensitivity ranged from 27.7% [27] to 100% [30], specificity from 13% [26] to 89.1% [28], positive predictive value (PPV) from 4.1% [28] to 59% [25] and negative predictive value (NPV) from 67% [26] to 97.5% [28].

The choice of a higher anti-dsDNA ab threshold (>300 IU/ml vs 50–300 IU/ml) led to higher specificity (89.1% vs 57.1% for mild/moderate flares) and lower sensitivity (28.4% vs 51.8% for mild/moderate flares) [27, 28].

Data concerning the delay between the elevation of anti-dsDNA ab and subsequent flares were not always available. When available, they were heterogeneous, ranging from once per month [7, 9, 13, 17, 24] to every 6 weeks [4, 11, 12], every 3 or 4 months [6, 8, 15, 16, 20, 22, 23, 27, 28], every 6 months [10, 14] and up to 1 year or 18 months [18, 26]. In addition to data concerning delays, those concerning the amount of increase of anti-dsDNA ab titres were frequently missing [5, 6, 8–12, 14, 19, 20, 22, 23]. The threshold most frequently chosen to define a significant rise was an increase greater than 25% of the preceding value [7, 13, 16, 26, 29].

#### Complement and complement split products

Complement and/or complement split products were analysed in 19 studies [6–10, 12, 19, 20, 23, 27, 28, 31–38] (Table 2). The first study assessing the predictivity of complement consumption in SLE flares was conducted by Lloyd and Schur in 1981 [19], and reports the importance of complement depression before exacerbations. Low baseline complement levels could be associated with subsequent SLE flares according to seven studies [7–10, 35, 36, 38] but these results were not consistent with each other, depending on the complement fraction studied (C3 and/or C4 and/or CH50): C3 was found to be associated with flares in four studies [7, 9, 10, 36], whereas C4 was found to be associated in three studies [8, 10, 35] and CH50 in two studies [7, 38]. The occurrence of complement decrease during the course of the disease as revealed by serial measurements was associated with a subsequent flare in two studies [12, 32], whereas three other studies did not prove such an association [6, 20, 34]. Persistently low C3 was predictive of renal flares in two independent studies [10, 23].

**Table 2:** Predictivity of complement in SLE flares

Complement fraction(s)	Number of patients (*n*)	Findings	Study
Positive results			
C3, C4 and CH50	57	Increased incidence of exacerbation in patients with decreased level of C3 or CH50	Tomioka et al., 2008 [7]
C3 and C4	562	Low C3 level (< 900 mg/L) was an independent predictor of a severe SFI flare	Petri et al., 2013 [9]
C3 and C4	299	Low C3 and C4 were risk factors for a later A or B flare in the mucocutaneous, renal and haematologic systems	Petri et al., 2009 [10]
C3	32	Low baseline serum C3 (< 900 mg/L) was a predictor for shorter time to flare	Ng et al., 2007 [36]
C3 and C4	46	Baseline C4 titres were low (< 10 mg/dl) in a significantly higher percentage of relapsing patients	El Hachmi et al., 2003 [8]
C3 and C4	145	C4 level (< 11 mg/dl) was a significant prognostic factor for renal flares	Illei et al., 2002 [35]
CH50	60	CH50 level was an independent predictor of lupus flares	Viallard et al., 2001 [38]
C1q, C3, C4, C5 and C9	143	Decrease of C4, followed by decreases of C1q and C3 levels (< 40% of normal values), started 25 to 20 weeks before renal involvement	Swaak et al., 1986 [12]
C3a, C5a, C3 and C4	40	C3a levels rose significantly (>200 ng/ml) 1–2 months prior to flare	Hopkins et al., 1988 [32]
C3	189	Persistently low C3 level was a predictor of nephritic renal flares	Mok et al., 2004 [23]
C1q, C3, C4	27	When patients were clinically active, mean values of C1q, C4 and CH50 were the lowest obtained for these markers	Lloyd and Schur, 1981 [19]
C3 and C4	71	Lower C4 levels (< 12 mg/dl), but not C3 levels, significantly predicted renal flares	Birmingham et al., 2010 [37]
C3 and C4	218	For renal flares: low C3 (0.5–0.74 g/L), sensitivity 34.8%, specificity 63.1%; low C4 (0.1–0.13 g/L), sensitivity 19.4%, specificity 79%	To et al., 2011 [27]
C3 and C4	218	For severe flares: low C3 (0.5–0.74 g/L), sensitivity 29.2%, specificity 63%, PPV 2.3%, NPV 96.7%; low C4 (0.1–0.13 g/L), sensitivity 19.2%, specificity 79%, PPV 2.8%, NPV 96.0%	To et al., 2011 [28]
C3, C4, CH50 and complement split products: Ba, Bb; C4d; SC5b-9	86	Most sensitive marker of flare: elevated C4d (> 8.5 mg/ml). Highest specificity and greatest predictive value for flare: elevated Bb (> 1.2 mg/ml)	Buyon et al., 1992 [33]
Negative results			
C3, C4 and C1q	202	Fluctuations were poor predictors of exacerbations	Esdaile et al., 1996 [20]
C3 and C4	53	Decreasing complement levels did not precede changes in disease activity	Ho et al., 2001 [34]
CH50	120	No consistent association of complement titre with flares in the subsequent year	Mirzayan et al., 2000 [6]
C3, C4 and CH50	73	No difference between patients who flared and patients who did not	Walz LeBlanc et al., 1994 [31]

*NPV* negative predictive value, *PPV* positive predictive value, *SFI* SLE Flare Index, *SLE* systemic lupus erythematosus

Results were expressed in terms of sensitivity, specificity and predictive values in four studies [27, 28, 33, 37] (Additional file 4). The results were heterogeneous: decreased C3 sensitivity ranged from 28.7% [27] to 45% [33], and decreased C3 specificity ranged from 63.1% [27] to 87.5% [28]. Decreased C4 sensitivity ranged from 19.1% [28] to 64.0% [33], and decreased C4 specificity ranged from 45.0% [33] to 79.0% [27]. CH50 sensitivity and specificity were evaluated only once, with the respective results of 71.0% and 29.0% [33]. Assessments of NPV were always satisfactory, with values superior to 95% (ranging from 95.8% for low C4 [28] to 98.3% for very low C3 [28]).

Some complement split products (C3a, C4d, Ba, Bb, SC5b9) were found to be informative in predicting lupus flares, particularly C3a (1–2 months prior to disease flare, C3a levels increased significantly for all 10 patients studied who experienced flares later), C4d (highest sensitivity 86.0%) and Bb (highest specificity 81.0%) [32, 33].

#### Anti-C1q antibodies

Authors reported very good NPV for lupus nephritis [30, 39, 40], ranging from 97.0% (95% CI 88.0–99.0%) [40] to 100.0% [30, 39]. For instance, in one study, none of the 50 patients with negative anti-C1q antibodies developed any sign of renal involvement during follow-up (median duration 24 months, range 1–60 months) [39]. NPV was less impressive in one study (70.0%) [29] (Table 3). PPV was always unsatisfactory (ranging from 50 to 56%). The high NPV of anti-C1q antibodies, especially for nephritis [30, 39], seemed to be of particular interest, suggesting that the occurrence of severe nephritis is quite improbable in the absence of anti-C1q antibodies. These results seemed promising for clearly identifying patients who are at low risk for flares or renal involvement.

**Table 3:** Predictivity of anti-C1q antibodies in SLE flares

Number of patients (*n*)	Findings	Study
68	For renal flares: sensitivity 71%, specificity 92%, PPV 50%, NPV 97%	Siegert et al., 1993 [40]
151	10 of 14 patients who developed proliferative nephritis had significant increases in anti-C1q level	Coremans et al., 1995 [15]
151	NPV 100% for nephritis	Marto et al., 2005 [39]
70	For severe lupus nephritis: sensitivity 100%, specificity 95.7%, PPV 50%, NPV 100%	Meyer et al., 2009 [30]
23	For renal flares: sensitivity 75.7%, specificity 84%, PPV 56%, NPV 70%	Matrat et al., 2011 [29]

All anti-C1q antibodies were measured using enzyme-linked immunosorbent assays

*NPV* negative predictive value, *PPV* positive predictive value, *SLE* systemic lupus erythematosus

#### Anti-nuclear antibodies, antibodies against extractable nuclear antigens and antibodies against nucleosomes

Antibodies against extractable nuclear antigens (anti-ENA) and anti-nucleosomes were studied in eight reports [5, 6, 9, 22, 36, 41–43] (Table 4). Associations between anti-ENA and the occurrence of a flare were found in six studies, with the important limitation that these results were reported in only one study each, and none of them has been reproduced: anti-nuclear antibodies (ANA) [6], baseline anti-ENA [36], anti-Sm [5, 9], anti-histone [22] and anti-nucleosome [42]. No correlations with disease activity were found with anti-Ro [41, 43], anti-La, anti-Sm and anti-ribonucleoprotein (anti-RNP) [41]. Repetition of the measurement of anti-ENA antibodies appeared not to be useful in assessing disease activity in SLE, and the determination of anti-ENA antibody profiles should be limited to the diagnosis period.

**Table 4:** Predictivity of anti-ENA in SLE flares

Biomarker(s)	Number of patients (*n*)	Findings	Study
Anti-Sm ab, ANA	110	Presence of anti-Sm ab at baseline was found at a higher incidence in patients developing exacerbation(s)	Swaak et al., 1989 [5]
ANA	120	ANA titre was associated with flares in the subsequent year	Mirzayan et al., 2000 [6]
ANA, anti-nucleosome and anti-histone ab	199	Patients with anti-histone ab (≥ 3 SDs above control mean) at baseline had a higher risk of developing lupus nephritis	Cortes-Hernandez et al., 2004 [22]
Anti-nucleosome ab	21	Time to first flare was significantly correlated with the presence of anti-nucleosome and high anti-nucleosome ab titres	Ng et al., 2006 [42]
Anti-ENA	32	Baseline anti-ENA was an independent predictor of flare	Ng et al., 2007 [36]
ANA, anti-Sm ab	562	Anti-Sm positivity (≥ 15 units/ml) at baseline predicted flares	Petri et al., 2013 [9]
Anti-Ro	47	Fluctuations of anti-Ro/SS-A ab levels were not predictive of flares	Praprotnik et al., 1999 [43]
Anti-Ro, anti-La, anti-Sm and anti-RNP ab	45	Fluctuations of anti-Ro, anti-La, anti-Sm and anti-RNP were not associated with flares	Agarwal et al., 2009 [41]

*ab* antibody, *ANA* anti-nuclear antibodies, *anti-RNP* anti-ribonucleoprotein, *anti-ENA* anti-extractable nuclear antigen, *SD* standard deviation, *SLE* systemic lupus erythematosus

#### Circulating immune complexes

Two reports [18, 19], published in 1980 and 1981, studied the associations of circulating immune complexes with the occurrence of flares. In the study by Abrass et al. [18], circulating immune complexes were measured by both solid-phase (SC1q) and fluid-phase C1q (FC1q) binding assays. An increase in SC1q binding assay results correctly predicted a change in the manifestations of SLE 82% of the time. In comparison, changes in FC1q binding assay failed to predict a change in disease activity correctly. In the other study, immune complexes were measured by C1q binding assay C1qBA and ADCC (antibody-dependent cell-mediated cytotoxicity) inhibition assay [19]. Only 50% of the patients had increased levels of C1qBA prior to clinical exacerbation. These tests are no longer used in clinical practice.

#### Erythrocyte sedimentation rate and C-reactive protein

No statistically significant association between change in erythrocyte sedimentation rate (ESR) between two visits and a future change in disease activity was found [38, 44]. In another study, ESR elevations were associated with flares [6].

Petri et al. [9] demonstrated that, according to univariate analysis, elevated C-reactive protein (CRP) at baseline predicted SLE flares by three indices (BILAG, Safety of Estrogens in Lupus Erythematosus National Assessment–Systemic Lupus Erythematosus Disease Activity Index (SELENA-SLEDAI), SLEDAI Flare Index (SFI)) during the course of the study, but this association was no longer persistent in multivariate analysis.

### Predictors of flares: experimental and newly developed biological markers, a new hope?

#### Cytokines, chemokines and their receptors

Several cytokines and chemokines or their soluble receptors were examined in 14 studies [9, 45–57] (Table 5). The ability of B-lymphocyte stimulating factor (BLyS), also known as B-cell activating factor from the TNF family (BAFF), to predict a subsequent SLE flare was dismissed in two studies [47, 52] but confirmed in two others [9, 48]. Three studies revealed an increase in the plasma levels of soluble IL-2R or sCD25 (which is the alpha chain of IL-2R) prior to disease exacerbation [52, 55, 57], while another study revealed a higher expression of CD25 on the surface of circulating lymphocytes [56]. The results concerning other cytokines, chemokines and receptors were single reports; consequently, generalization of these data did not seem suitable.

**Table 5:** Predictivity of cytokines and chemokines in SLE flares

Cytokines or chemokines	Number of patients (*n*)	Findings	Study
BAFF/BLyS	42	Changes in BAFF levels were unrelated to disease flares	Becker-Merok et al., 2006 [47]
BAFF/BLyS	245	Increase in BLyS level was associated with the occurrence of mild-to-moderate flares	Petri et al., 2008 [48]
BAFF/BLyS	562	Baseline BLyS level independently predicted flare	Petri et al., 2013 [9]
BLyS, APRIL and 50 analytes: innate and adaptive cytokines, chemokines and soluble TNFR superfamily members	28	Patients with impending flare had significant alterations in the levels of 27 soluble mediators at baseline	Munroe et al., 2014 [52]
CCL2 (MCP-1), CCL19 (MIP-3B), CXCL10 (IP-10)	267	Patients with high baseline chemokine levels were at increased risk for flares	Bauer et al., 2009 [49]
CXCL2, CXCL10	25	High CXCL10 and CXCL2 were predictive of increased disease activity	Andrade et al., 2012 [50]
IL-2R	26	Activation of T cells occurs prior to clinical disease activity	Spronk et al., 1996 [56]
sIL-2R	NA	Levels of sIL-2R rose significantly up to the moment of maximal disease activity	Spronk et al., 1994 [57]
sCD25	3	Levels of sCD25 increased preceding periods of exacerbations	Swaak et al., 1995 [55]
IFN-α, IFN-γ-inducible protein 1 and sialic acid-binding Ig-like lectin 1	79	None of the investigated biomarkers was a predictive variable for flares	Rose et al., 2013 [51]
Soluble IL-7 receptor (sIL-7R)	105	High sIL-7R levels associated with renal flares	Lauwerys et al., 2014 [53]
IL1-RA, TNFRI	41	Patients who flared had higher baseline plasma levels of IL-1RA and TNFRI	Guthridge et al., 2014 [54]

*APRIL* a proliferation-inducing ligand, *BAFF* B-cell activating factor, *BLyS* B-lymphocyte stimulator, *CCL* chemokine ligand, *CXCL* chemokine (C–X–C motif) ligand, *s* soluble, *SLE* systemic lupus erythematosus, *TNFR* tumour necrosis factor receptor type

#### Expression of specific markers by T cells

Five studies [38, 55–58] assessed the relationship between the expression of specific antigens or specific transcription factors by T cells and disease flares. Markers testifying to the activation of T lymphocytes were the most studied, by measurement of serum levels of specific activation antigens or by flow cytometry. Levels of sCD27 increased before exacerbation in the three patients studied [55]. HLA-DR expression by CD8^+^ T lymphocytes [38] or by CD4^+^ lymphocytes [56] appeared to be associated with the occurrence of a lupus flare. Expression of CD25 was also considered a marker of lymphocyte activation, and the results achieved were presented in the preceding section [56].

Another study examined the expression of the specific transcription factor FoxP3 in different subsets of CD4^+^ T cells (naïve T-regulatory (Treg) cells, effector Treg cells and FoxP3^+^ non-Treg cells) in a small cohort of SLE patients [58]. Most of the patients who developed flares had anomalies in FoxP3^+^CD4^+^ T-cell subsets before flares (the most prevalent anomaly observed before flares was an increase in FoxP3^+^ non-Treg cells), while those who maintained the absence of anomalies did not develop flares.

#### Markers of endothelial activation

Three cellular adhesion molecules, required for cell-to-cell interactions, were evaluated in two studies [59, 60]: the results were contradictory regarding soluble vascular cell adhesion molecule-1 (sVCAM-1) in the two reports and were clearly negative for soluble intercellular adhesion molecule-1 (sICAM-1) and soluble E-selectin (sE-selectin).

#### Urinary markers

Seven records studied biomarkers in the urine of SLE patients [61–67]. Five molecules, namely tumour necrosis factor-like weak inducer of apoptosis (TWEAK), macrophage colony-stimulating factor (M-CSF), neopterin, regulated on activation, normal T-cell expressed and secreted (RANTES) and urinary neutrophil gelatinase-associated lipocalin (uNGAL), were measured in urine by ELISA (or by reverse-phase high-performance liquid chromatography for neopterin) in five studies [63–67]. These markers were all positively correlated with subsequent SLE renal flares. TWEAK seemed of particular interest because the results were consistent through three different studies, and this marker is considered a potentially promising therapeutic target for lupus nephritis [68]. While other reports evaluated single molecules measured in urine, two studies assessed the expression of transcription factors or the transcriptional expression of cytokines [61, 62]. One study evaluated the expression of T-bet by urinary sediment cells and revealed that a high urinary T-bet expression level was an independent predictor of a lupus flare [61]. In the other study, a significant increase was found in the mRNA levels of monocyte chemotactic protein (MCP)-1 and FoxP3 before disease flares, along with decreases in IL-17 and GATA-3 [62].

#### Other experimental biomarkers

In 1991, ter Borg et al. [69] evaluated the ability of anti-70-kDa and anti-A polypeptides antibodies to predict SLE flares but failed.

Plasma adiponectin did not change significantly before flares, whereas longitudinal testing revealed that urine adiponectin increases began in the 2 months prior to renal flares [70].

Plasma cell peaks (CD27^++^, CD20^–^ cells) preceded the increase in disease activity [71].

Patients with circulating anti-dsDNA ab-secreting cells had significantly lower cumulative rates for remaining disease flare-free than patients without these cells in the circulation [72]. Nearly all of the patients with circulating anti-dsDNA ab-secreting cells relapsed within 12 months.

## Discussion

Despite the clinical interest in and numerous publications on biomarkers in SLE, there is no validated and widely accepted biomarker for flare prediction in SLE to date. In this systematic review, none of the newly studied biomarkers stood out, and the routinely performed biomarkers appeared to be deceiving, with contradictory results. Data concerning some biomarkers, such as anti-C1q antibodies, BLyS or TWEAK, seemed promising and could be useful in identifying SLE patients who are at high risk for flares and especially at high risk for renal disease, but these results require confirmation in larger studies. Clinicians must be aware that, at this time, none of these biological markers is completely reliable in diagnosing exacerbations, and none of them can be considered a serologic gold standard. The use of some laboratory parameters, such as anti-dsDNA ab, complement and anti-C1q, and their close follow-up are still considered the most powerful tools in predicting disease flares, even if limited. Thus, they are included in the current EULAR recommendations [73] and should not be abandoned easily.

This systematic review was, to the best of our knowledge, the first aiming to compile all of the available data on biomarkers predicting SLE flares. The strengths of this study included a comprehensive review of the reports on predictive biomarkers in SLE with well-defined inclusion criteria, performed with the help of a research librarian and with data extraction performed by two independent reviewers.

Our conclusions must be considered in the presence of possible limitations. One of the main limitations of this work was the high heterogeneity between the study designs, reflecting the heterogeneity of the disease itself. The best study design to emphasize the predictivity of a biomarker is a prospective study. Nevertheless, 14 studies were retrospective and might have been biased. Another design issue was follow-up frequencies: patients were either observed monthly [13, 17] or every 3 months [20, 22]. The risk of missing an increase in antibody levels is negligible with monthly measurements. Therefore, studies with monthly follow-ups might detect correlations more often. However, the clinical utility of monthly measurements has not yet been assessed, and the economic burden of close monitoring must be justified. The study populations were heterogeneous by ethnicity, time from diagnosis, sample size, treatment and disease activity at baseline. Above all, the heterogeneity in flare definitions and disease activity measurements was the most important limiting factor and could prevent comparison of different studies. The concept of a flare in this disease is very complex, and there is no universally accepted definition to date. The absence of a standardized definition complicates the interpretation and comparison of findings. Clinicians use many indices (SLEDAI, SELENA-SLEDAI, BILAG, Physician Global Assessment (PGA), SFI, European Consensus Lupus Activity Measurement (ECLAM)), which, although valid and sensitive, do not evaluate the disease in the same manner [74]. None of them has emerged yet as a gold standard, which led to inconsistent results depending on the index used [17]. Flare rates, which are different between reports, can also vary within a report depending on the index used [9]. The choice to include different degrees of severity (mild/moderate or severe) might have modified the total number of flares and thus affected the sensitivity and specificity of biomarkers. Concerning the biomarkers themselves, the assay techniques could first lead to heterogeneity due to their different performance characteristics. The sensitivity, specificity, PPV and NPV were different if the Farr assay, *C. luciliae* assay or ELISA was used for measuring anti-dsDNA ab [25, 26, 29, 30]. The Farr radioimmunoassay is believed to detect high-avidity antibodies, *C. luciliae* assays detect antibodies of intermediate avidity and ELISA detects both high-avidity and low-avidity antibodies [75, 76]. We could not determine with certainty whether one of these assays is more performant than another due to the low number of studies. The results can also vary according to the class of Ig considered for the measurement of anti-dsDNA ab (positive results with IgG and negative results with IgM) [16], which could explain, in part, the discrepancies in the results between studies. Moreover, the use of plasma or serum samples for the assay can be important: some authors believe that it is necessary to use plasma instead of serum to measure anti-dsDNA ab to avoid the possible binding of antibodies to DNA from disrupted blood cells [76]. Finally, the threshold levels chosen for positive test results could also be a source of discrepancy. To increase the ability of biomarkers to predict flares, some authors have combined traditional ones. Values (especially PPV) obtained in this manner were often higher than those of each marker obtained separately [28–30], but these data are scarce. Development of prediction models for outcomes of the disease using multiple biomarkers that can be measured at the same time with commercial kits would actually be of great interest [77] and a study of the transcriptome profile could also be promising [78]. Last but not least, it is of particular interest to underline the concept of “serologically active, clinically quiescent” (SACQ) SLE, with discordance between clinical and serologic features, which adds another level of complexity for the prediction of flare in some patients. A large international task force reached recent consensus on the definition of SACQ, which corresponds to the presence of anti-dsDNA ab and/or hypocomplementemia [79]. In this group of patients, fluctuations in anti-dsDNA ab and complement levels cannot predict flares and no consensus was obtained by the task force regarding the definition of remission in those patients [79].

Clinicians need more data to help them to choose the correct biomarker or biomarker combination to predict flares, and the quest for a predictive marker of disease activity should be a major focus of SLE clinical research. With the advent of personalized medicine, with an increasing number of targeted therapies, reliable non-invasive predictors of flares are of great interest. There is a need to conduct prospective studies with standardized guidelines about severity indices and flare definitions to validate potentially relevant biomarkers and to bring them into the field of daily clinical practice, in the same manner as has been performed for therapeutic trials [73].

To homogenize study patterns, we propose conducting multicentre, longitudinal, prospective, controlled studies, including patients with SLE who fulfil the revised ACR criteria and who are ethnically diverse. The most appropriate SLE flare index must be chosen among BILAG, SLEDAI or ECLAM, as encouraged by EULAR recommendations [73], and only one score should be used. Follow-up visits should occur every 3 months over at least 3 years. Thresholds for increases and decreases in each biological marker should be defined clearly. Biomarkers should be validated with assessments of sensitivity, specificity and predictive values. Candidate biomarkers with promising results in small patient cohorts must to be validated in large populations. Biomarker panels must be developed.

## Conclusions

No conclusions could be drawn from this systematic review due to the lack of standardized data. Efforts should be undertaken to optimize future research on potential SLE biomarkers to develop validated candidates. Thus, we propose a standardized pattern for future studies.

## Additional files

Additional file 1: Presenting search strategies. (DOC 201 kb)

Additional file 2: Study characteristics. (DOC 300 kb)

Additional file 3: Anti-dsDNA antibody sensitivity, specificity, PPV and NPV. (DOC 43 kb)

Additional file 4: Complement sensitivity, specificity, PPV and NPV. (DOC 47 kb)

## Abbreviations

ACR: American College of Rheumatology
ANA: Anti-nuclear antibodies
Anti-dsDNA ab: Anti-double-stranded DNA antibodies
Anti-ENA: Antibodies against extractable nuclear antigens
Anti-RNP: Anti-ribonucleoprotein
BAFF: B-cell activating factor from the TNF family
BILAG: British Isles Lupus Assessment Group
BLyS: B-lymphocyte stimulating factor
CRP: C-reactive protein
ECLAM: European Consensus Lupus Activity Measurement
ESR: Erythrocyte sedimentation rate
EULAR: European League Against Rheumatism
MCP: Monocyte chemotactic protein
M-CSF: Macrophage colony-stimulating factor
NPV: Negative predictive value
PGA: Physician Global Assessment
PPV: Positive predictive value
PRISMA: Preferred Reporting Items for Systematic Reviews and Meta-Analyses
RANTES: Regulated on activation, normal T-cell expressed and secreted
SACQ: Serologically active, clinically quiescent
SELENA-SLEDAI: Safety of Estrogens in Lupus Erythematosus National Assessment–Systemic Lupus Erythematosus Disease Activity Index
sE-selectin: Soluble E-selectin
sICAM-1: Soluble intercellular adhesion molecule-1
SLE: Systemic lupus erythematosus
sVCAM-1: Soluble vascular cell adhesion molecule-1
Treg: T-regulatory
TWEAK: Tumour necrosis factor-like weak inducer of apoptosis
uNGAL: Urinary neutrophil gelatinase-associated lipocalin
