# Supplementary material for: Predictive biological markers of systemic lupus erythematosus flares: a systematic literature review
Source: Arthritis Res Ther. 2017 Oct 24;19:238. doi: 10.1186/s13075-017-1442-6 (PMC5655881; doi:10.1186/s13075-017-1442-6)
Supplement: Supplementary file 1 — Presenting search strategies. (DOC 201 kb) [file 13075_2017_1442_MOESM1_ESM.doc]

**Additional file 1. Search strategies**

Pubmed:

(("biological markers"[MeSH Terms] OR "biological markers"[All Fields]) AND "lupus erythematosus, systemic"[MeSH Terms] AND ("severity of illness index"[MeSH Terms] OR Flare[All Fields] OR Flares[All Fields] OR exacerbation[All Fields] OR exacerbations[All Fields] OR "predictive value of tests"[Mesh] OR "Disease Progression"[MeSH Terms])) OR ("lupus erythematosus, systemic"[MeSH Terms] AND ("exacerbations"[Title] OR "flares"[title] OR "exacerbation"[Title] OR "flare"[Title]) AND (English[lang] OR French[lang])))

Scopus:

(TITLE-ABS-KEY(systemic lupus erythematosus) AND TITLE-ABS-KEY(biological markers) OR TITLE-ABS-KEY(biological marker) AND (TITLE-ABS-KEY(flare) OR TITLE-ABS-KEY(flares) OR TITLE-ABS-KEY(exacerbation) OR TITLE-ABS-KEY(exacerbations) OR TITLE-ABS-KEY(disease progression) OR TITLE-ABS-KEY(predictive value of tests) OR TITLE-ABS-KEY(severity of illness index))) AND ( LIMIT-TO(LANGUAGE,"English" ) OR LIMIT-TO(LANGUAGE,"French" ) )
