# Supplementary material for: Predictive biological markers of systemic lupus erythematosus flares: a systematic literature review
Source: Arthritis Res Ther. 2017 Oct 24;19:238. doi: 10.1186/s13075-017-1442-6 (PMC5655881; doi:10.1186/s13075-017-1442-6)
Supplement: Supplementary file 2 — Study characteristics. (DOC 300 kb) [file 13075_2017_1442_MOESM2_ESM.doc]

**Additional file 2: Studies characteristics**

| **Study** | **Study design** | **Age mean/median (years) (range)** | **Sex (% females)** | **Ethnicity (%)** |
| --- | --- | --- | --- | --- |
| Swaak et al, 1979 [4] | Longitudinal | NA | NA | NA |
| Swaak et al, 1989 [5] | Prospective | Mean = 47 ± 17 (Men)  = 37 ± 15 (Women) | 85.4 | NA |
| Mirzayan et al, 2000 [6] | Prospective | Mean = 45.2 (18–69) | 88.3 | 100% Caucasian |
| Tomioka et al, 2008 [7] | Prospective | Mean = 40.2 ± 17.2 | 91.2 | NA |
| El Hachmi et al, 2003 [8] | Prospective | NA | NA | NA |
| Petri et al, 2013 [9] | Retrospective | Mean = 38.1 ± 12.0 | 93.0 | 48% White, 9% African, 21% Asiatic |
| Petri et al, 2009 [10] | Prospective | Mean = 47 | 88.0 | 57% Caucasian, 37% African American, 3% Asian, 2% Hispanic, and 1% other ethnicities |
| Swaak et al, 1982 [11] | Prospective | NA | NA | NA |
| Swaak et al, 1986 [12] | Prospective | NA | NA | NA |
| ter Borg et al, 1990 [13] | Prospective | Mean = 37.5 (18-73) | 84.7 | NA |
| Oelzner et al, 1996 [14] | Retrospective | Mean = 39 (15 - 64) | 86.8 | NA |
| Coremans et al, 1995 [15] | Prospective | Renal relapses: 28.9 ± 10.2. Nonrenal relapses: 34.3 ± 10.1. Inactive disease: 42.6 ± 10.7. | 86.0 | NA |
| Bootsma et al, 1997 [16] | Prospective | Median = 36 (17–75) | 91.2 | 88.2% White, 0% Black, 11.8% Orientals |
| Ho et al, 2001 [17] | Prospective | NA | 98.1 | 41% White, 55% Black, 4 % Other ethnic groups |
| Abrass et al, 1980 [18] | Prospective | From 17 to 63 years | 91.6 | 56.0% Black, 25.0% White, 12.5% Mexican-American, 6.5% Other ethnicity |
| Lloyd et al, 1981 [19] | Retrospective | NA | 85.1 | NA |
| Esdaile et al, 1996 [20] | Prospective | Median = 36 (12-78) | 86.0 | 84.0% White, 7.4% Black |
| Steiman et al, 2010 [21] | Retrospective | NA | NA | NA |
| Cortés-Hernandez et al, 2004 [22] | Prospective | NA | Without nephritis: 87.0 With nephritis: 79.0 | NA |
| Mok et al, 2004 [23] | Retrospective | Mean = 31.1 ± 10.7 | 88.4 | 100% Chinese |
| Linnnik et al, 2005 [24] | Retrospective and prospective | Trial: Abetimus: Mean = 37.2 ± 10.8. Placebo: Mean = 34.6 ± 10.0. | Trial 1: Abetimus: 90.2, Placebo: 92.8. | Trial 1: Abetimus: 35.9% White, 37.0% Black, 27.2% Other. Placebo: 45.4% White, 27.8% Black, 26.8% Other. Trial 2: Abetimus: 47.6% White, 22.8% Black, 29.7% Other. Placebo: 44.4% White, 28.1% Black, 27.5% Other. |
|  |  | Trial 2: Abetimus: Mean = 37.1 ± 11.1 Placebo: Mean = 35.2 ± 9.9. | Trial 2: Abetimus: 87.6, Placebo: 86.3. |  |
| Pan et al, 2014 [25] | Prospective | Mean = 37.2 (+/- 15.5) | 95.5 | 50% White, 32% African American, 9% Asian, 9% Other |
| Hillebrand et al, 2014 [26] | Retrospective | Patients with exacerbations: 37.2 (18.8–62.3).  Patients without exacerbations: 50.2 (17.4–70.3) | 89.6 | 87.5% White, 6.25% Black, 6.25% Asian |
| To et al, 2011 [27] | Retrospective | NA | NA | NA |
| To et al, 2011 [28] | Retrospective | NA | NA | NA |
| Matrat et al, 2011 [29] | Longitudinal-retrospective | Median = 28 (17–54) | 91.2 | NA |
| Meyer et al, 2009 [30] | Retrospective | Active lupus with nephritis: Median = 30 (19-58).  Active lupus without nephritis: Median = 28 (17-48).  Inactive lupus without nephritis: Median = 35 (20-76). | 91.4 | Active lupus with nephritis: 86.7% Caucasian, 13.3% Black, 0% Asian.  Active lupus without nephritis: 60.6% Caucasian, 27.2% Black, 12.1% Asian. Inactive lupus without nephritis: 86.3% Caucasian, 13.6% Black, 0% Asian. |
| Walz LeBlanc et al, 1994 [31] | Prospective | Mean = 33 years at diagnosis | 93.2 | NA |
| Hopkins et al, 1988 [32] | Prospective | NA | NA | NA |
| Buyon et al, 1992 [33] | Prospective | NA | NA | NA |
| Ho et al, 2001 [34] | Prospective | NA | 98.1 | 41% White, 55% Black, 4% Other ethnic groups |
| Illei et al, 2002 [35] | Retrospective | Complete response: Median = 29.8  Partial response/stabilization: Median = 27,1 | Complete response: 88.0 Partial response/stabilization: 89.0 | Complete response: 67% Caucasian, 22% African American, 7% Hispanic, 4% Asian. Partial response/stabilization: 63% Caucasian, 26% African American, 6% Hispanic, 6% Asian. |
| Ng et al, 2007 [36] | Prospective | Mean = 34 (21–54) | 93.6 | 53% White, 28% African Caribbean, 9% Asian, 9% Other |
| Birmingham et al, 2010 [37] | Prospective | NA | NA | NA |
| Viallard et al, 2001 [38] | Prospective | Mean = 37.95 | 93.3 | 95.0% Caucasian, 3.3% African, 1.6% Asian |
| Marto et al, 2005 [39] | Retrospective | Median = 44 (18-74) | 94.0 | NA |
| Siegert et al, 1993 [40] | Prospective | Mean = 38 (14-75) | 95.6 | NA |
| Agarwal et al, 2009 [41] | Prospective | Mean = 43 (25 - 69) | 91.0 | 33% White, 44% Black, 16% Asian, 1 of Hispanic origin and 2 patients of mixed race |
| Ng et al, 2006 [42] | Retrospective | Mean = 45 | 95.2 | 57% White, 14% African Caribbean, 19% Asian, 9.5% Other |
| Praprotnik et al, 1999 [43] | Prospective | Mean = 31.5 (18-54) | 93.8 | 100% Caucasian |
| Stojan et al, 2012 [44] | Prospective | NA | NA | NA |
| Spronk et al, 1992 [45] | Prospective | Mean = 39.2 (15-70) | 83.1 | NA |
| Tokano et al, 1999 [46] | Retrospective | 23–37 | 92.3 | NA |
| Becker-Merok et al, 206 [47] | Retrospective | Mean = 39.7 | 92.0 | NA |
| Petri et al, 2008 [48] | Prospective | Mean = 41.5 | 92.0 | 67% White, 31% African American, 2% Asian |
| Bauer et al, 2009 [49] | Prospective | Mean = 42 (+/- 12) | 87.0 | 56% North Americans of European descent, 37% African Americans, 7% other ethnicity |
| Andrade et al, 2012 [50] | Prospective | Mean = 48 +/- 10 | 84.0 | 72% Caucasian, 28% African-American |
| Rose et al, 2013 [51] | Prospective | Mean = 36 (20 - 69) | 89.9 | 89.9% Caucasian, 10.1% Asian, 0% Black |
| Munroe et al, 2014 [52] | Prospective | Flare SLE: 46.9 +/-14.0  Non-flare SLE: 47.2 +/-12.3  Healthy controls: 46.8+/- 13.5 | 100% | 100% Caucasian |
| Lauwerys et al, 2014 [53] | Prospective | NA | NA | NA |
| Guthridge et al, 2014 [54] | Prospective | NA | NA | NA |
| Swaak et al, 1995 [55] | Prospective | Mean = 33 | 100.0 | NA |
| Spronk et al, 1996 [56] | Prospective | Mean = 34 (21– 55) | 88.5 | NA |
| Spronk et al, 1994 [57] | Prospective | Mean = 31.2 (21– 47) | 83.1 | NA |
| Miyara et al, 2011 [58] | Prospective | NA | NA | NA |
| landolt-Marticorena et al, 2013 [59] | Prospective | NA | NA | NA |
| Spronk et al, 1994 [60] | Prospective | Mean = 32 (20 – 50) | 86.3 | NA |
| Chan et al, 2007 [61] | Prospective | Mean = 38.8 +/-11.2 | 95.0 | NA |
| Szeto et al, 2012 [62] | Prospective | Flare group: Mean = 38.1 +/-9.6  Control group: Mean = 39.4 +/-9.4 | 89.4 | NA |
| Tian et al, 2007 [63] | Prospective | Without renal flare: Mean = 24.9 +/-3.7  With renal flare: Mean = 24.4 +/-2.8 | Without renal flare: 91.2 With renal flare: 86.6 | NA |
| Rubinstein et al, 2010 [64] | Prospective | Mean = 41.0 (16-67) | 91.0 | 47% Hispanic, 46% African American, 2% White, 4% others |
| Wisniacki et al, 2013 [65] | Prospective | Mean = 46.0 (+/-12) | 92.0 | 59% Caucasian, 34% African American |
| Schwartz et al, 2009 [66] | Prospective | AECOM: Mean = 36.0 (31 - 44)  OSS: Mean = 30 (28 - 36) | AECOM: 80.0 OSS: 100.0 | AECOM: 58% African American, 38% Hispanic, 3% Caucasian OSS: 38% African American, 61% Caucasian |
| Lim et al, 1994 [67] | Prospective | Mean = 43 (+/-12.8) | 95.6 | NA |
| ter Borg et al, 1991 [69] | Prospective | Mean = 32.9 (15-70) | 83.0 | NA |
| Rovin et al, 2005 [70] | Prospective | Non-renal flare: Mean = 33.7 ± 2.7. Renal flare: Mean = 33 ± 1.9. | Patients who flared: 90.7 | Patients who flared: 67.4% Caucasian, 32.6% African American |
| Boekel et al, 2011 [71] | Prospective | NA | NA | NA |
| Hanaoka et al [72] | Prospective | Presence of anti-dsDNA antibody-secreting cells: 44.2 ± 10.7.  Absence of anti-dsDNA antibody-secreting cells: 45.1 ± 15.8. | Presence of anti-dsDNA antibody-secreting cells: 58  Absence of anti-dsDNA antibody-secreting cells: 86 | NA |

NA: Not available
