# Supplementary material for: Predictive biological markers of systemic lupus erythematosus flares: a systematic literature review
Source: Arthritis Res Ther. 2017 Oct 24;19:238. doi: 10.1186/s13075-017-1442-6 (PMC5655881; doi:10.1186/s13075-017-1442-6)
Supplement: Supplementary file 3 — Anti-dsDNA antibody sensitivity, specificity, PPV and NPV. (DOC 43 kb) [file 13075_2017_1442_MOESM3_ESM.doc]

**Additional file 3. Anti-dsDNA antibodies sensitivity, specificity, positive and negative predictive values**

| **Flare type** | **Assay** | **Sensitivity (%)** | **Specificity (%)** | **PPV (%)** | **NPV (%)** | **Study** |
| --- | --- | --- | --- | --- | --- | --- |
| Severe SELENA-SLEDAI flares | CLIF | 62.0 | 80.0 | 59.0 | 81.0 | Pan et al, 2014 [25] |
| Severe nephritis (class IV or V) | ELISA | 100.0 | - | - | - | Meyer et al, 2009 [30] |
| NA | Farr | 86.0 | 13.0 | 32.0 | 67.0 | Hillebrand et al, 2013 [26] |
|  | EliA | 71.0 | 57.0 | 43.0 | 81.0 |  |
| Renal flares | Farr | 72,7 | 77.0 | 53.0 | 72.0 | Matrat et al, 2011 [29] |
| Mild/moderate flares | NA | PdsDNA* 51.8 | 57.1 | 5.1 | 96.4 | To et al, 2011 [28] |
|  | NA | HPdsDNA** 28.4 | 89.1 | 10.3 | 96.5 |  |
| Severe flares | NA | PdsDNA* 56.1 | 57.1 | 4.1 | 97.5 |  |
|  | NA | HPdsDNA** 26.0 | 88.8 | 7.1 | 97.3 |  |
| Renal flares | NA | PdsDNA* 61.1 | 57.0 | NA | NA | To et al, 2011 [27] |
|  | NA | HPdsDNA** 27.7 | 88.6 | NA | NA |  |

| *PdsDNA: Positive anti-dsDNA antibodies (50-300 IU/ml)  **HPdsDNA: Highly Positive anti-dsDNA antibodies (>300 IU/ml)  CLIF: *Crithidia luciliae* immunofluorescence; ELISA: Enzyme-linked immunosorbent assay; EliA: automated enzyme fluoroimmunoassay  PPV: Positive Predictive Value. NPV: Negative Predictive Value.  NA: Not Available. |
| --- |
