# Supplementary material for: Predictive biological markers of systemic lupus erythematosus flares: a systematic literature review
Source: Arthritis Res Ther. 2017 Oct 24;19:238. doi: 10.1186/s13075-017-1442-6 (PMC5655881; doi:10.1186/s13075-017-1442-6)
Supplement: Supplementary file 4 — Complement sensitivity, specificity, PPV and NPV. (DOC 47 kb) [file 13075_2017_1442_MOESM4_ESM.doc]

**Additional file 4. Complement sensitivity, specificity, positive and negative predictive values**

| **Flare type** | **Biomarker** | **Sensitivity (%)** | **Specificity (%)** | **PPV (%)** | **NPV (%)** | **Study** |
| --- | --- | --- | --- | --- | --- | --- |
| NA | Decreased C3 | 45.0 | 74.0 | Predictive value: 48.0 |  | Buyon et al, 1992 [33] |
|  | Decreased C4 | 64.0 | 45.0 | Predictive value: 38.0 |  |  |
|  | Decreased CH50 | 71.0 | 29.0 | Predictive value: 41.0 |  |  |
| Renal flare | C3 | 70.0 | 73.0 | 22.0 | 97.0 | Birmingham et al, 2010 [37] |
|  | C4 | 49.0 | 74.0 | 17.0 | 95.0 |  |
| Renal flares | Low C3* | 28.7 | 63.1 | NA | NA | To et al, 2011 [27] |
|  | Very Low C3** | 57.4 | 87.1 | NA | NA |  |
|  | Low C4° | 19.4 | 79.0 | NA | NA |  |
|  | Very Low C4°° | 55.3 | 73.6 | NA | NA |  |
| Mild/moderate flares (SELENA-SLEDAI flare instrument) | Low C3* | 34.8 | 63.2 | 3.7 | 95.9 | To et al, 2011 [28] |
|  | Very Low C3** | 43.1 | 87.5 | 12.3 | 97.4 |  |
|  | Low C4° | 19.1 | 78.9 | 3.7 | 95.8 |  |
|  | Very Low C4°° | 49.1 | 74.0 | 7.4 | 97.2 |  |
| Severe flares (SELENA-SLEDAI flare instrument) | Low C3* | 29.2 | 63.0 | 2.3 | 96.7 |  |
|  | Very Low C3** | 51.1 | 87.4 | 11.0 | 98.3 |  |
|  | Low C4° | 19.2 | 79.0 | 2.8 | 96.9 |  |
|  | Very Low C4°° | 52.9 | 73.9 | 5.9 | 98.0 |  |

*Low C3: 0.5 0.74g/l

**Very Low C3: <0.5g/l

°Low C4: 0.1-0.13g/l

°°Very Low C4<0.1g/l

PPV: Positive Predictive Value

NA: Not available
